# Immediate effects of a lumbar spine manipulation on pain sensitivity and postural control in individuals with nonspecific low back pain: a randomized controlled trial

**DOI:** 10.1186/s12998-020-00316-7

**Published:** 2020-06-03

**Authors:** Jefferson Fagundes Loss, Luciano de Souza da Silva, Iã Ferreira Miranda, Sandro Groisman, Edgar Santiago Wagner Neto, Catiane Souza, Cláudia Tarragô Candotti

**Affiliations:** 1 Universidade Federal do Rio Grande do Sul, Escola de Educação Física, Fisioterapia e Dança, Felizardo, 750 – LAPEX Building, Porto Alegre, RS 90690-200 Brazil; 2 Instituto Brasileiro de Osteopatia, Porto Alegre, Brazil; 3 Faculdade Social da Bahia, Salvador, Brazil

**Keywords:** Non-specific low back pain, HVLA lumbar manipulation, Pain sensitivity, Numerical pain rating scale, Algometry, Postural parameters

## Abstract

**Background:**

According to the American Physical Therapy Association, there is strong evidence to show that vertebral mobilization and manipulation procedures can be used to improve spinal and hip mobility and reduce pain and incapacity in low back pain patients that fit the clinical prediction rule. Objectives: To evaluate the immediate effects of high-velocity low-amplitude (HVLA) manipulation on pain and postural control parameters in individuals with nonspecific low back pain.

**Methods:**

This study used a participant-blinded and assessor-blinded randomized controlled clinical trial involving a single session, in which 24 participants were randomly distributed into control (simulated manipulation) and intervention (HVLA lumbar manipulation) groups. The primary (pain: subjective pain intensity and pressure pain threshold) and secondary outcomes (postural control: ellipse area, center of pressure [COP] excursion, COP RMS velocity, and differences between the COP and center of projected gravity) were evaluated before and after the session using a numerical pain scale, algometer, and a force platform. For all outcomes, multiple mixed 2 (group) × 2 (time) ANOVAs were performed.

**Results:**

For the subjective pain intensity, only time was significant as a main effect, where pre-intervention presented a greater value then post-intervention (F [1.44] = 4.377; *p* = 0.042; *r* = 0.30). For the pressure pain threshold no significant effect was found. For the postural control parameters, as a main effect, only the ellipse area was significantly greater in the control group (F [1.44] = 6.760; *p* = 0.013; effect size = 0.36).

**Conclusions:**

There was a reduction in subjective pain intensity, evaluated using a numerical scale, in both the intervention and control groups immediately after the intervention, suggesting that the spinal manipulation had a similar effect to the placebo procedure. No effect of HVLA lumbar manipulation was identified for postural control variables in either the intervention or control groups.

**Trial registration:**

The study was registered at ClinicalTrials.gov under the number NCT02312778, registered at 14 September 2014.

## Introduction

Back pain affects the lives of millions of people and is a considerable financial burden for Western health care systems [1]. Thus, in many countries evidence-based clinical guidelines have been issued to standardize the management of low back pain [2]. The diagnostic and therapeutic recommendations contained in these guidelines are generally similar. However, there are some discrepancies for recommendations regarding spinal manipulation for low back pain.

The Orthopedic Section of the American Physical Therapy Association (APTA) is seeking to create evidence-based practice guidelines for the orthopedic physical therapy management of patients with musculoskeletal impairments [3]. According to APTA, there is strong evidence to show that vertebral mobilization and manipulation procedures can be used to improve spinal and hip mobility and reduce pain and incapacity in low back pain patients that fit the clinical prediction rule [4]. According to a recent systematic review, spinal manipulation produces similar effects to other therapies (i.e. non-drug: exercise; and drug treatments: non-steroidal anti-inflammatory drugs, analgesics) recommended for chronic low back pain, and it is better than non-recommended interventions (i.e. non-effective: light soft tissue massage, no treatment, waiting list control; and potentially even harmful treatments: electrotherapies) [5].

Back pain has been associated with altered motor control in the spinal erector muscles [6–8] and impaired proprioception in the spine [9, 10], hindering lumbar stability, body balance, posture, and postural control. Impaired detection of passive motion is seen in people with low back pain. This may be related to changes in the excitatory threshold of mechanoreceptors [10], which could explain these postural control deficits [11]. There is evidence that subjects with low back pain experience greater center of pressure (COP) oscillation [6, 11–15], suggesting that this pain affects the neuromuscular responses required to maintain balance. However, in a systematic review focused on the effects of manual therapy, the authors showed that there is little research to support the use of manual therapy for the improvement of postural stability [16]. They concluded that new controlled trials are needed to provide robust clinical evidence of whether manual therapy and manipulation play a role in improving postural stability and balance.

Traditionally, controlled trials that evaluate the effects of manipulations on pain have been assessed based on scales and/or instruments that measure the level of pain [17] and local hyperalgesia [18]. It has been theorised that spinal manipulation also acts on the excitatory threshold of the mechanoreceptors [1], which are fundamental for maintaining balance and posture [16], it seems possible that the effects of manipulation may be assessed based on COP variations. Hence, the aim of the present study is to evaluate the immediate effects of high-velocity low-amplitude (HVLA) lumbar manipulation on pain and COP in subjects with low back pain (LBP). We hypothesized that vertebral manipulation would reduce both pain levels and COP oscillation.

## Methods

### Trial design

The present study is reported in accordance with the TIDieR recommendations [19]. This HVLA lumbar manipulation in subjects with LBP study is a randomized controlled clinical trial involving a control group (CG) and intervention group (IG) with a 1:1 allocation rate. It was conducted between January and March of 2015 with the approval of the Ethics Committee of the Universidade Federal do Rio Grande do Sul (number 834.848/CAEE 36001414.2.0000.5347) and registered at ClinicalTrials.gov (NCT02312778), according to the CONSORT 2010 [20].

### Eligibility criteria

The participants were recruited through social media and newspapers as well as in physiotherapy clinics. Manual therapy studies use the clinical prediction rule to select and classify homogenous groups of participants that may benefit from the use of spinal manipulation as a therapeutic intervention [4, 21–24]. The spinal manipulation clinical prediction rule has five criteria: pain duration of less than 16 days, no symptoms distal to the knee, score less than 19 in the fears and beliefs questionnaire, spinal stiffness, and internal rotation of the hip greater than 35 degrees [4].

The inclusion criteria for the study were men and women between the ages of 20 and 60 years with daily or almost daily lumbar pain in the previous 3 months [25] and who met at least four of the five clinical prediction criteria for HVLA lumbar manipulation [4, 23]. The exclusion criteria were the presence of radiating lower back pain, neurological alteration in the lower limbs (sensitivity, muscle force, and/or patellar or Achilles reflexes), previous surgery, medical diagnosis of spondylolisthesis, spinal stenosis, inflammatory disease, cancer, lower limb musculoskeletal degenerative diseases, pregnancy, pathologies and/or medications that may affect balance, osteopenia and/or osteoporosis, and women over 50 who had not had a bone densitometry exam.

### Intervention

Prior to intervention, the most hypomobile vertebra within the L1 to L5 vertebral segment was identified in all the members of both groups, using the clinical posterior-anterior vertebral pressure test applied with the subjects in ventral decubitus. Testing was performed on the spinous processes of the vertebrae. The osteopath placed the hypothenar eminence of the hand over the spinous process of the vertebra to be tested and applied gentle but firm pressure on the spinous process. By doing so, the stiffness of each vertebra was judged as either normal, hypomobile, or hypermobile. Thus, the most hypomobile vertebra was identified by comparing the mobility of the vertebrae immediately above and below [4]. It is important to highlight that, while assessing for segmental hypomobility is common in manual therapy practice, this test has only fair inter- and intra-rater reliability (kappa <.40) [26].

**Fig. 1:**
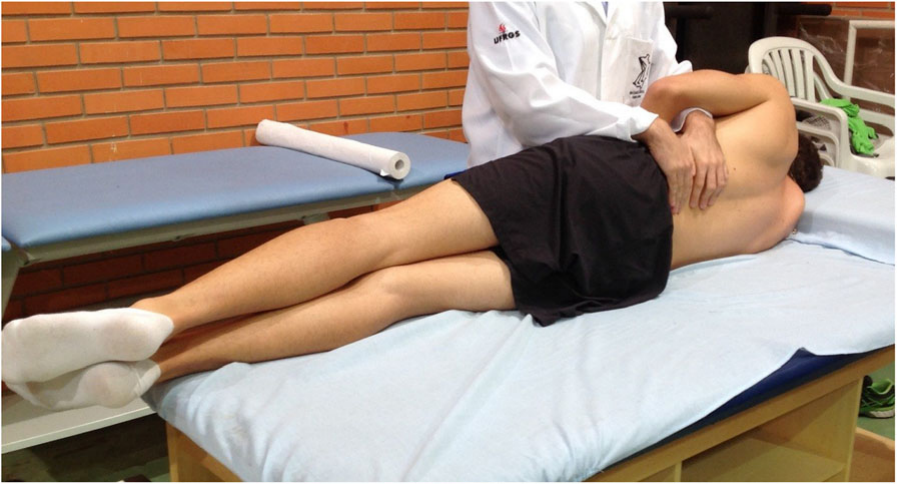
Initial position for both IG and CG

Each subject from both the CG and IG received a single intervention. All interventions were conducted on an examination table, with the subject in right lateral decubitus (Fig 1) because assessment on that side presents better intra- and inter-reproducibility [27, 28]. All interventions were conducted by an osteopath with 3 years of experience, who had been trained to identify vertebral mobility and perform the spinal manipulation (HVLA).

**Fig. 2:**
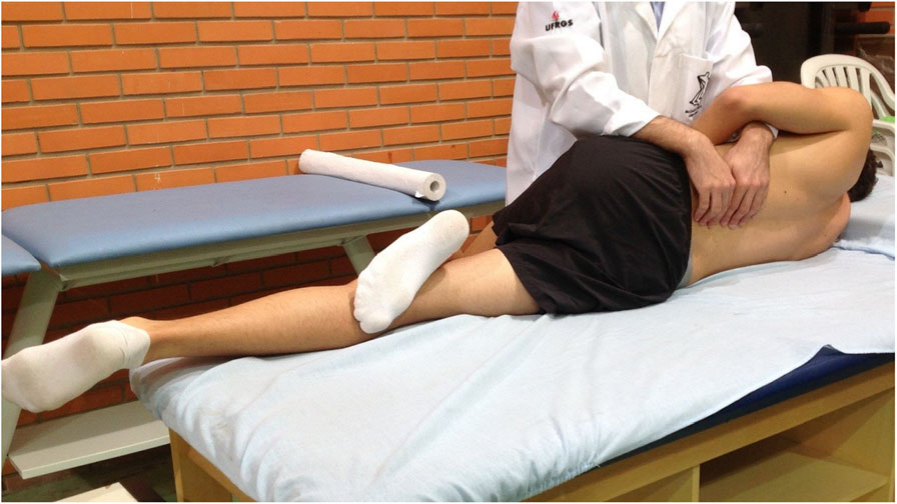
Final position for simulated manipulation of CG participants

**Fig. 3:**
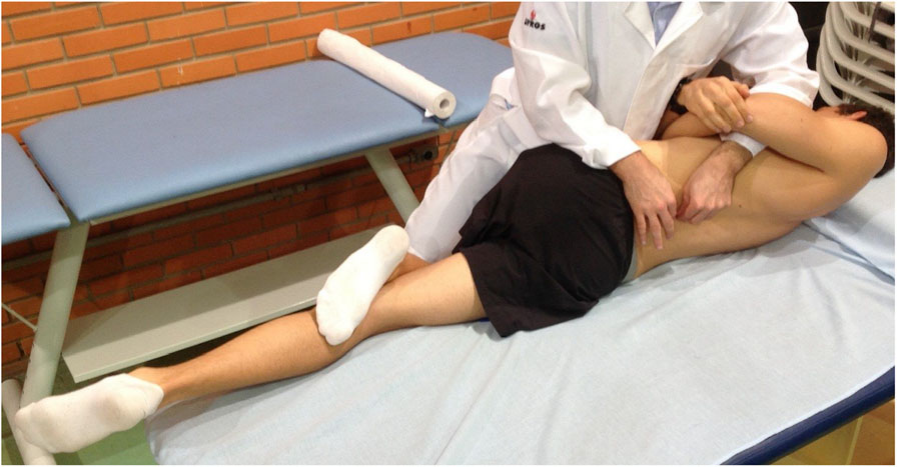
Final position for manipulation of IG participants

The participants allocated to the CG received simulated manipulation with no intended therapeutic effect. The participants allocated to this group were positioned in the right lateral decubitus position with the left leg flexed at the hip and knee and the left foot resting in the right popliteal fossa without stretching the paravertebral tissues (Fig 2). The participant remained in the position for approximately 20 s without receiving the HVLA thrust, which is the average time required to carry out HVLA lumbar manipulation [29]. For the IG, the HVLA lumbar manipulation was conducted according to Gibbons and Tehan [30], by locating the hypomobile vertebra when performing the thrust (Fig 3). During the manipulation it was unnecessary to produce an audible ‘pop’ [31].

### Assessment of outcomes

All assessments were performed by the same assessor, following the same sequence, namely: numerical pain scale (subjective pain intensity), algometer (pressure pain threshold) and test on force platform (postural outcomes).

**Fig. 4:**
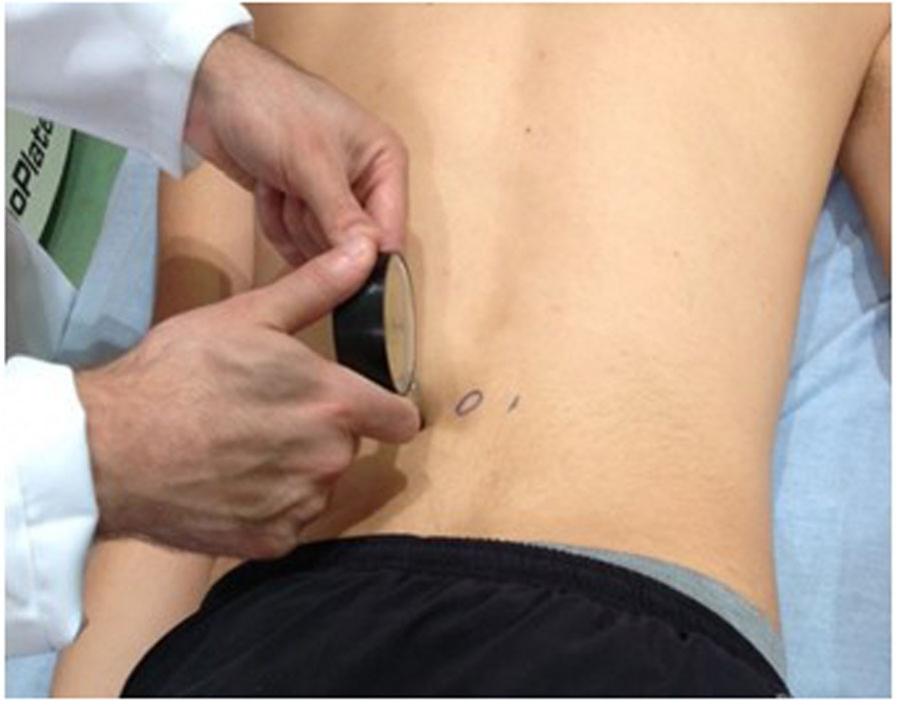
Pressure pain threshold assessment

A self-reported 10-point-scale [32, 33] was used to assess the subjective pain intensity at rest. Pressure pain threshold (PPT) was assessed using a 10 kgf analogic pressure algometer (Wagner Instruments, Greenwich, CT-USA). The PPT was assessed on three sites: the lumbar spinal processes of the most hypomobile vertebra identified during the posterior-anterior vertebral pressure test; and bilaterally on the spinal erector muscle group, located 5 cm each side of the lumbar spinal processes [34]. The assessment was carried out with the participants lying in the prone position (Fig. 4), in accordance with the procedures used by Oliveira [34]. In short, the assessor pressed the algometer at a rate of approximately 0.5 kgf/s. Participants were asked to say “pain” when the sensation of pressure or discomfort became a clear sensation of pain. Three measurements were collected for each site at 30-s intervals. The average of three measures was used for data analysis. If the participant did not report pain at a force equivalent to 10 kgf, the test was interrupted and this value was considered the PPT. Prior to the assessment, the assessor performed 2 demonstrations of the procedure on the extensor muscles of the dominant forearm to ensure that the participant understood the test. While no information was found regarding the amount of difference between two assessments that should be considered clinically important using the analogic pressure algometer, van der Roer et al. [35] recommended that when using a self-reported 10-point-scale a difference of 2.5 points is clinically important.

Considering the possibility that the excitatory threshold of the mechanoreceptors is influenced by spinal manipulation [1], a secondary outcome, postural control was assessed in two ways: based on the COP and the Center of Projected Gravity (COPG). A BTS P-6000 force platform (BTS Bioengineering, Milan-Italy) with a sampling frequency of 500 Hz was used to evaluate the COP.

To evaluate the COPG, the SMTS DX high-definition motion capture system (BTS Bioengineering, Milan-Italy), consisting of 10 infra-red cameras with a sampling frequency of 500 Hz and a spatial resolution of 4 megapixels, was used. First, the barycenter of four reflective markers placed on the anterior and posterior superior iliac spines were used to estimate the center of gravity. This method was adapted from the sacral method, which is used for walking [36, 37]. Then, the COPG was calculated by projecting the three-dimensional coordinates of the center of gravity onto the floor. The COP and COPG signals were smoothed using a fourth-order low-pass Butterworth filter with a cut-off frequency of 4 Hz [38]. The variables for postural control were [11]:

- The difference between the COP and COPG curves, found using the Root Mean Square (RMS) value: this analysis represents the close relationship between the COP and COPG curves in the Anterior-Posterior (AP) and Mid-Lateral ML directions during semi-static erect posture.
- Ellipse area: defined as the area representing the dispersion of the COP positions in the AP and ML directions, where 95% of the data (COP positions) are present [39]. The area represents the degree of stability of the subject; the larger the area, the lower the stability.
- Total pressure center excursion: defined as the total distance travelled by the COP over a given time in the AP and ML directions [40].
- RMS velocity: defined as the square root of the quadratic mean of the COP displacements divided by the time between two successive positions, in both the AP and ML directions [40].

### Study procedures

The collection environment was controlled in order to avoid sound or visual stimuli that could affect the evaluation of the COP. The researcher reviewed the procedural instructions with each participant. In accordance with the guidelines from Zok et al. [41], the participants were instructed to *“stand as still as possible and stare at the fixed target in front of you”* while in the orthostatic position on the force platform, with their feet approximately shoulder-width apart and their arms resting at the side of the body. The subjects from both groups were asked to adopt a semi-static upright posture, which was held for 30 s, eyes open with the gaze fixed on an x-shaped marker placed 3 m in front of the platform at a height of 1.75 m from the ground.

The procedure was repeated three times, generating three curves both before and after the intervention for each group. The initial analyses were performed based on Curve 2, arbitrarily. If any problems were encountered when collecting Curve 2 (i.e. unwanted participant movements, noising signal, etc) then Curve 1 was used. If necessary, Curve 3 was used as the last option. If such problems persisted, the participant would be excluded.

### Sample size

When calculating the sample size, the subjective pain intensity was taken into account based on data from de Oliveira et al. [34], who also investigated the immediate effects of manipulative therapy in patients with low back pain. Using the G-Power software version 3.1.7 (Universität Kiel, Germany), the *t*-test family (means: difference between two dependent means – matched pairs), means and standard deviations pre- (6.07 ± 2.12) and post- (4.16 ± 2.34) intervention, and assuming a correlation between groups of 0.5, an effect size of 0.85 was determined. Thus, assuming an alpha of .05 and power (1-β) of 0.80, a total of 10 subjects per group was necessary. Assuming a loss of 20%, we decided on 12 subjects per group.

### Randomization

The group randomization was simple and was generated using Random Allocation Software (Informer Technologies, Inc.) by a researcher uninvolved in the subsequent research stages. This same researcher distributed the labels in sealed opaque envelopes, respecting the numbering on each label.

### Blinding

This study was participant-blinded and assessor-blinded. The participants did not know to which group they had been allocated. They were informed there would be two different interventions and that they would be offered an alternative intervention after the end of the study if they wished. Nobody asked for an alternative intervention. However, the participant-blinding was not formally assessed. The evaluators rating the variables were also blinded because they did not know which intervention the participants had received, since they were not present at the time of the individual interventions.

### Study design

The first stage consisted of recruiting individuals and having them sign the consent form. Then, one of the researchers completed an assessment form containing items such as the participant’s age, body mass, height (Parisian point equivalent to 0.66 cm), level of physical activity (considered when executed with guidance from a health professional and at least three time a week), and study eligibility criteria, including the clinical prediction rule.

In the second stage, the primary and secondary outcomes were evaluated prior to beginning the intervention. The third stage, consisting of the post-randomization intervention, was always performed by the same osteopath without the presence of any of the other researchers. The post-intervention evaluation was performed during the fourth stage. There was no interval between stages. The time between pre-intervention evaluation and intervention, and between intervention and post-intervention evaluation was just enough for the respective researchers to leave and enter the room.

### Statistical methods

The data normality was assessed using the Kolmogorov–Smirnov test. If the data distribution was normal, parametric tests were applied to analyze the data. For the variables with non-parametric distribution, transformations were performed (log [x], sqrt [x], 1/x, etc.). SPSS 22.0 software (IBM) was used with a significance value of 5%.

The descriptive variables were presented as the mean and standard deviation with a confidence interval of 95%. For all outcomes, multiple mixed two-way ANOVAs were performed, with the group (control or intervention) as the independent factor and time (pre- or post-intervention) as the repeated factor. Given that the statistical test for the primary outcome was different from that in the original pre-register, the G-Power software was used to calculate the posthoc power (1 – β). Effect size (r) was calculated using the square root of the partial-squared eta (sum of squares of the effect divided by the total sum of the squares of the evaluated effects plus the sum of the squares of the error of the evaluated effect) and was classified as small when r ≤ 0.10, average when 0.10 < r 0.30, and large when r = 0.50 [42, 43].

## Results

**Fig. 5:**
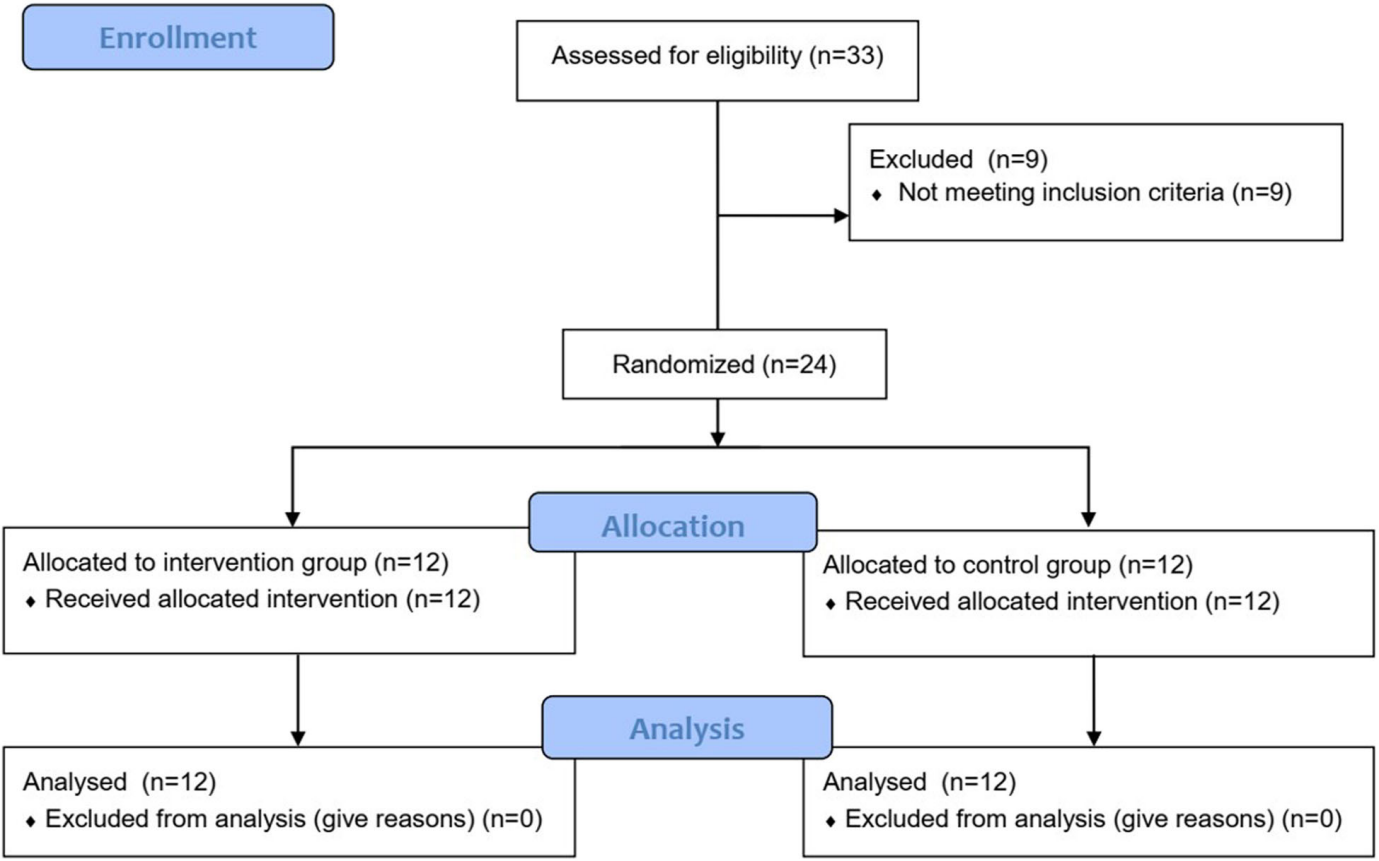
Study flowchart

Of the first 33 individuals who were contacted and agreed to participate, nine were excluded, four because they presented radiating pain and five because of age. Thus, 24 individuals participated, 12 in the IG and 12 in the CG, with no sample losses during the survey (Fig 5). There was no statistically significant difference between-group (*p* ≥ 0.05) at pre-intervention according to the sample description data (Table 1).

**Table 1:** Sample characteristics

Variable	CG	IG	*t* Statistic (df)	*P* value
Gender	50%	50%	0.00 (22)	1.00
Age (years)	43.9 ± 9.6	41.7 ± 12.8	0.46 (22)	0.64
Body mass (kg)	73.7 ± 12.1	73.6 ± 11.4	0.01 (22)	0.98
Height (m)	1.72 ± 0.11	1.69 ± 0.10	0.66 (22)	0.51
Foot size (Parisian point)	39.1 ± 3.0	38.7 ± 2.8	0.27 (22)	0.78
Physical activity	12%	15%	−1.25 (22)	0.22

*CG* Control Group, *IG* Intervention Group, *df* Degree of Freedom

When the subjective pain intensity was analyzed (Table 2) a significant time main effect was observed, where pre-intervention presented a greater value than post-intervention (F [1.44] = 4.377; *p* = 0.042; r = 0.30). There was no significant effect for the group factor (F [1.44] = 0.369; *p* = 0.547; *r* = 0.09) or for group*time interaction (F [1.44] = 0.223; *p* = 0.639; *r* = 0.07). The power (1 – β) was calculated as 96.0, 15.4 and 11.0% for time, group and group*time respectively.

**Table 2:** Comparison of pain (primary outcome) before and after intervention

Outcome	Group	Mean pre- (standard deviation)	Mean post- (standard deviation)	Post-Pre (95% IC)
**Subjective pain intensity**	Control	3.8 (± 2.7)	2.8 (± 2.6)	−1.0 (− 3.3; 1.3)
	Intervention	4.5 (± 1.6)	2.9 (± 1.2)	−1.6 (−2.8; − 0.4)
**Pressure pain threshold** **(Spinal Process)**	Control	7,0 (± 2,5)	6,8 (± 2,3)	−0.2 (−2.2; 1.9)
	Intervention	6,1 (± 2,3)	6.5 (± 2,6)	0.4 (−1.7; 2.5)
**Pressure pain threshold** **(Right Erector)**	Control	7.2 (± 2.3)	6.8 (± 2.5)	−0.4 (−2.4; 1.7)
	Intervention	6.8 (± 2.6)	7.3 (± 2.5)	0.5 (−1.7; 2.6)
**Pressure pain threshold** **(Left Erector)**	Control	7.1 (± 2.3)	7.1 (± 2.3)	0.0 (−1.9; 1.9)
	Intervention	6.8 (± 2.9)	7.3 (± 2.6)	0.5 (−1.8; 2.8)

Regarding the pressure pain threshold (Table 2), there was no significant effect for the group factor, time factor, or group*time interaction, in the lumbar spinal processes, on the left side or on the right side of the spinal erector muscle group. The power (1 – β) ranged between 5 and 27%.

When the COP was analyzed through the ellipse area (Table 3), a significant group main effect was observed, where the CG presented a larger area (F [1.44] = 6.760; *p* = 0.013; r = 0.36). There was no significant effect for the time factor (F [1.44] = 0.461; *p* = 0.501; r = 0.10) or for group*time interaction (F [1.44] = 0.567; *p* = 0.455; r = 0.11). Regarding the total COP excursion, COP RMS velocity, and differences between the COP and COPG in the AP and ML directions (Table 3), there was no significant effect for the group factor, time factor, or group*time interaction.

**Table 3:** Comparison of the postural parameters (secondary outcome) before and after intervention

Outcome	Group	Mean pre- (standard deviation)	Mean post- (standard deviation)	Post-Pre (95% IC)
**Area of the ellipse (cm**^**2**^**)**	Control	2.2 (± 1.6)	1.7 (± 1.3)	−0.5 (−0.7; 1.7)
	Intervention	1.1 (± 0.9)	1.1 (± 0.8)	0.0 (−0.7; 0.7)
**COP displacement (cm)**	Control	44.3 (± 10.2)	42.5 (± 7.9)	−1.8 (−9.5; 6.0)
	Intervention	40.7 (± 7.9)	41.5 (± 8.0)	0.8 (−5.9; 7.6)
**COP RMS Velocity (cm/s)**	Control	2.7 (± 0.3)	2.7 (± 0.4)	0.0 (−0.3; 0.3)
	Intervention	2.5 (± 0.3)	2.5 (± 0.3)	0.0 (−0.2; 0.2)
**Difference between COP and COPG in AP (cm)**	Control	1.0 (± 0.7)	1.2 (± 0.7)	0.2 (−0.3; 0.8)
	Intervention	1.0 (± 0.6)	0.8 (± 0.4)	−0.2 (− 0.7; 0.2)
**Difference between COP and COPG in ML (cm)**	Control	0.6 (± 0.6)	0.8 (± 0.8)	0.2 (−0.5; 0.8)
	Intervention	0.6 (± 0.4)	0.5 (± 0.3)	−0.1 (− 0.4; 0.2)

*COP* Center Of Pressure, *RMS* Root Mean Square, *COPG* Center Of Projected Gravity, *AP* Anterior-Posterior, *ML* Medium-Lateral

## Discussion

The results show a significant reduction in subjective pain intensity for both the control and intervention groups when comparing pre- and post-intervention, with no difference between-groups. Nevertheless, the difference in subjective pain intensity was less than the recommended 2.5 points considered necessary for clinical importance [35]. Furthermore, the reduction in subjective pain intensity for the CG suggests the manipulation effect is similar to the placebo effect, which disagrees with the results reported by Rubinstein et al. [5] The reduction in subjective pain intensity found in the CG may be attributed to a placebo effect or possibly explained by the Hawthorne effect [44, 45]. Regarding the IG, corroborating reports have shown that there is a hypoalgesic effect immediately after HVLA manipulation in the lumbar region [46, 47].

Regarding pressure pain threshold, there was no significant increase in the pain threshold for either the IG or CG, which is similar to the results reported by Dorron et al. [48], confirming that manipulation would not have an immediate effect. However, Dorron et al. [48] did find significant differences in relation to the baseline when the pressure pain threshold was assessed 10, 20, and 30 min after the manipulation, with the pain threshold increasing up to 12%. Additionally, investigating different manipulations (large amplitude, small amplitude, and quasi-static) in asymptomatic subjects, Krouwel et al. [49] found an increase in the pain threshold immediately after manipulation, as did Millan et al. [50] who confirmed an effect of spinal manipulative therapy on the pressure pain threshold. However, in a recent systematic review, Aspinall et al. [51] reported that significant changes in the pressure pain threshold over time in low back pain populations are inconsistent. These results suggest that more studies are needed to understand the immediate effects of manipulation.

Regarding postural control, neither of the COP-related variables (ellipse area and total COP excursion) showed differences after the intervention (Table 3), which is consistent with results obtained by Goertz et al [52],. who found no changes in postural variables after lumbar manipulation. In our study there was a significant reduction in pain, which was not accompanied by a significant reduction in either the area of the ellipse or the COP velocity.

According to della Volpe et al., changes in postural strategy may underlie a dysfunction of the peripheral proprioceptive system or the central integration of proprioceptive information. Muscle pain may cause marked reduction of position sense, possibly through increased presynaptic inhibition of muscle afferents at the spinal level and/or by a down-regulation of cortical systems involved in proprioceptive processing [15]. Since our participants had pain and the subjective pain intensity was significantly reduced, we speculate that the pain level and/or the reduction in pain was insufficient to induce any alteration on postural variables. Furthermore, it is possible that our study was underpowered for the secondary outcome measures.

When the differences between the COP and COPG were analyzed in the pre- and post-intervention situations, there were no significant differences regardless of the group (Table 3). However, while on average there is no difference between the pre- and post-intervention evaluations, some individuals demonstrated considerable changes in relation to differences between the COP and COPG. This variable would seem to be worthy of greater attention.

### Study limitations

This study has several limitations. The first is related to the study design, which include only a single intervention and no follow-ups. Secondly, the limited sampling duration for the standing balance trials could be considered a potential study limitation. Thirdly, the participant-blinding was not formally assessed, which means there is no guarantee the participants were unaware the nature of the intervention they received (placebo or real). Another limitation refers to the sample size calculation, which only considered the subjective pain intensity. This fact may have under-powered our study for secondary outcome measures. Probably the main limitation is related to the sample size calculation, which we had assumed within-group comparisons. Thus, the results regarding the between-group comparisons are underpowered. In other words, our results regarding the lack of difference between groups need to be interpreted with caution (type II error possibility).

## Conclusion

HVLA lumbar manipulation in subjects with LBP showed no intervention-specific effects on subjective pain intensity or pressure pain threshold. However, there was a reduction in subjective pain intensity, in both the IG and CG immediately after the intervention, suggesting that the spinal manipulation had a similar effect to the placebo procedure. No effect was found for HVLA lumbar manipulation on postural control variables over time or as a function of group allocation, which raises the possibility that either HVLA does not influence posture or that this study was insufficiently powered to detect any effect.

## Supplementary information

**Additional file 1.**

**Additional file 2.**

## Data Availability

All data generated or analysed during this study are included in this published article [and its supplementary information files].
